# Preparation of High-Performance Composite Hydrogel Reinforced by Hydrophilic Modified Waste Rubber Powder

**DOI:** 10.3390/molecules26164788

**Published:** 2021-08-07

**Authors:** Rui Wu, Zuming Jiang, Zhenxing Cao, Zhaoyang Yuan, Yao Zhang, Lanlei Guo, Fuqing Yuan, Jinrong Wu, Jing Zheng

**Affiliations:** 1 State Key Laboratory of Polymer Materials Engineering, College of Polymer Science and Engineering, Sichuan University, Chengdu 610065, China; wurui641107971@stu.scu.edu.cn (R.W.); caozx1201@163.com (Z.C.); zhaoyangyuan@stu.scu.edu.cn (Z.Y.); 2 Exploration and Development Research Institute, Shengli Oilfield Company, SINOPEC, Dongying 257015, China; jzmedi@163.com (Z.J.); gaocai302@163.com (L.G.); gaocai20041@163.com (F.Y.); 3 Guangdong Provincial Key Laboratory of Naturel Rubber Processing, Agricultural Products Processing Research Institute of Chinese Academy of Tropical Agricultural Sciences, Zhanjiang 524001, China; zhang_yao@stu.scu.edu.cn

**Keywords:** rubber powder, modification, hydrogel, mechanical properties

## Abstract

In order to reduce the environmental pollution caused by waste rubber and to realize the recycling of resources, we proposed a facile method for the hydrophilic modification of waste rubber powder (HRP) and used it to reinforce a composite hydrogel. In the presence of toluene, dibenzoyl peroxide (BPO) diffused into the waste rubber powder. After the solvent was removed, BPO was adsorbed in the rubber powder, which was used to initiate the grafting polymerization of the acrylamide monomer on the rubber–water interface. As a result, the polyacrylamide (PAM) molecular chains were grafted onto the surface of the rubber powder to realize hydrophilic modification. The success of the grafting modification was confirmed by FTIR, contact angle testing, and thermogravimetric analysis. The hydrophilic modified waste rubber powder was used to reinforce the PAM hydrogel. Mechanical tests showed that the tensile strength and elongation at the break of the composite hydrogel reached 0.46 MPa and 1809%, respectively, which was much higher than those of pure PAM hydrogel. Such a phenomenon indicates that the waste rubber particles had a strengthening effect.

## 1. Introduction

With the development of the automobile industry, the amount of waste rubber produced is increasing every year. As a thermosetting elastomer, rubber usually takes hundreds of years to completely degrade. Landfills affect soil permeability and deteriorate the soil environment, while incineration releases massive harmful gas [1,2]. In order to reduce the environmental pollution caused by waste rubber, more and more people are beginning to pay attention to how to recycle these types of black waste [3,4]. Nowadays, the waste rubber powder (WRP) obtained from the crushing of waste rubber products is used as filler to prepare composite materials. On the one hand, it is conducive to the recycling of resources. On the other hand, it can reduce production costs. It has become one of the most promising methods for the treatment of waste rubber [5,6,7,8]. 

However, the unmodified WRP lacks the interfacial interaction with the matrix of other polymers, resulting in a decrease in the performance of the composite material, which greatly limits the recycling of WRP [9,10]. In order to improve the dispersion state and the compatibility of the particles with the matrix, a commonly used method is to graft polymer chains onto the surface of the particles. By adjusting the graft density (σ, chains/nm^2^) of the grafted molecular chain, the P/N ratio of the polymer molecular chain length (P) to the grafted molecular chain length (N) and the interaction between particles and the matrix changes so as to realize the control of mechanical properties [11,12]. Wu et al. found that a low graft density and a high molecular weight PLA grafted with SiO_2_ nanoparticles has a more obvious enhancement effect on the matrix, indicating that the interaction between the matrix chain and the long-grafted chain plays an important role in the performance of the composite [13].

Methods for modifying WRP include physical methods [14,15,16], chemical methods [17,18], and mechanochemical methods [19,20,21]. The physical method involves destroying the S-S cross-linking bond with radiation without destroying the C-C bond so as to cause surface of rubber powder desulfurization. According to reports in the literature, Liu et al. used waste rubber powder and ultrafine barium ferrite powder as raw materials to prepare a magnetic rubber composite material with high compatibility between the organic matrix and the inorganic dispersed phase by in situ modification in the microwave [22]. The chemical method usually uses reagents such as coupling agents and grafting agents to modify the surface of rubber powder. Du et al. used ultraviolet rays to treat rubber powder and grafted bismaleimide [23]. The results showed that the mechanical properties and elongation at the break of the blended rubber improved. The mechanochemical method involves using mechanical action to make the rubber powder react with chemical agents, thereby modifying the rubber powder. Wang et al. used 3-aminopropyltriethoxysilane (silane coupling agent KH550) as a surfactant to modify the surface of plasticized rubber powder (PRP) in order to improve its interface with epoxidized natural rubber [24].

As a high water-containing polymer with a three-dimensional cross-linked network structure, hydrogel is widely used in biomedicine [25,26,27], industry [28,29], agriculture, and in other fields [30,31,32] due to its unique properties. However, traditional synthetic hydrogels have the problems of the uneven distribution of the cross-linking points and the lack of energy dissipation mechanisms, resulting in poor mechanical properties, which greatly limit the application of hydrogels [33]. In order to solve this problem, researchers have innovatively developed a variety of hydrogels with excellent mechanical properties and novel structures, including nanocomposite hydrogels [34,35], homogeneous network hydrogels [36,37], and double network hydrogels [38,39]. Although these gels have relatively excellent properties, their fabrication methods are generally complex and costly. 

In this study, we used a simple method for the hydrophilic modification of the WRP and to introduce it into the hydrogel to prepare a WRP-reinforced composite hydrogel. The initiator BPO was diffused into the rubber powder particles through organic solvent swelling. Under heating, the BPO on the surface of the WRP initiated the polymerization of acrylamide monomer, and the polyacrylamide molecular chains were grafted on the surface to realize the hydrophilic modification of the rubber powder. The modified rubber powder was then added as an organic filler to the polyacrylamide hydrogel so that it could play a strengthening role. The results show that compared to pure polyacrylamide hydrogel, the mechanical properties of the composite hydrogel reinforced by modified WRP are much higher.

## 2. Results and Discussion 

### Preparation of HRP

**Figure 1:**
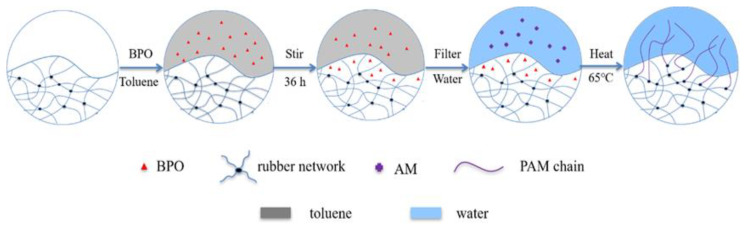
Schematic diagram of the HRP preparation.

In order to improve the hydrophilicity of WRP, we modified it by grafting PAM molecular chains to its surface. As shown in Figure 1, we soaked 4 g WRP in toluene with 7 g of dissolved initiator BPO. It was swelled because of the toluene, and the initiator BPO diffused into the molecular network with the solvent. On the one hand, by using such a large amount of BPO, , the soaking efficiency was very low; only about 10% of the BPO could enter the rubber network. On the other hand, the presence of antioxidants would hinder the progress of the polymerization reaction. After the solvent was removed, the WRP with the adsorbed initiator BPO was dispersed in an aqueous solution containing AM monomers. At 65 °C, BPO was used to initiate the polymerization of the AM monomers on the interface between the WRP and the aqueous solution. As a result, the PAM molecular chains were grafted onto the surface of the WRP, which greatly improved the hydrophilicity of the WRP. 

**Figure 2:**
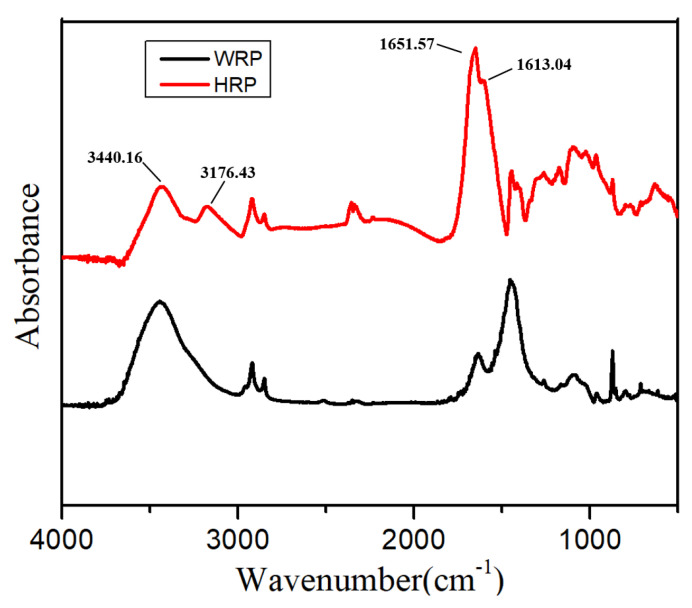
FTIR spectra of WRP and HRP.

The surface of PAM grafted WRP (HRP) was characterized by FTIR. Figure 2 shows the FTIR spectra of the modified and unmodified rubber powders. Compared to the WRP, the HRP had absorption peaks at 3440.16 cm^−1^ and 3176.43 cm^−1^, corresponding to the two absorption bands of the N-H stretching vibration. Additionally, the HRP manifested two absorption peaks at 1651.57 cm^−1^ and 1613.04 cm^−1^, attributed to the stretching vibration of C=O and the bending vibration of N-H, respectively. All of the above absorption peaks are characteristic peaks of the amide group. Collectively, these results indicate that the PAM chains were successfully grafted to the WRP.

**Figure 3:**
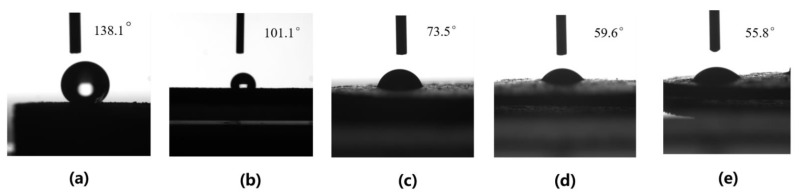
Contact angle of WRP (**a**), HRP_7-5_ (**b**), HRP_7-10_ (**c**), HRP_7-15_ (**d**), and HRP_7-20_ (**e**).

**Figure 4:**
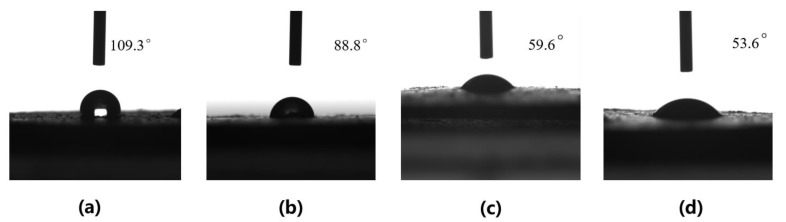
Contact angle of HRP_3-15_ (**a**), HRP_5-15_ (**b**), HRP_7-15_ (**c**), HRP_9-15_ (**d**).

In order to distinguish HRP prepared under different conditions, we renamed it to HRP_x-y_, where x and y represent the weight of the BPO and the AM added to the 4 g WRP, respectively. To study the effect of different amounts of AM on the hydrophilicity of HRP_7-y_, the contact angle of the HRP_7-y_ to water was measured. As shown in Figure 3, when the mass of the AM increased from 0 g to 15 g during grafting reaction, the contact angle of HRP_7-y_ decreased from 138.1° to 59.6°. A further increase in the amount of AM shows little change in the HRP contact angle. Additionally, the more AM that is present and the more PAM grafted onto the surface of the rubber powder, the higher the grafting ratio in Table 1. This result indicates that the hydrophilicity of HRP_7-y_ is significantly improved by grafting PAM chains, as the increasing the AM concentration within a certain range in the aqueous phase can evidently enhance the grafting efficiency. In addition, the amount of BPO has an impact on the hydrophilicity of HRP_x-15_, and the results are shown in Figure 4. With the increase of BPO from 3 g to 7 g, the contact angle gradually decreased from 109.3° to 59.6°. Similarly, as shown in Table 2, more BPO is beneficial to improve the grafting ratio. This indicates that the increase of BPO can initiate more AM monomer polymerization during the grafting reaction. When it is increased to 9 g, the quality of BPO does not have much influence on the contact angle, possibly because the adsorption of the BPO by the rubber powder has reached saturation. 

**Figure 5:**
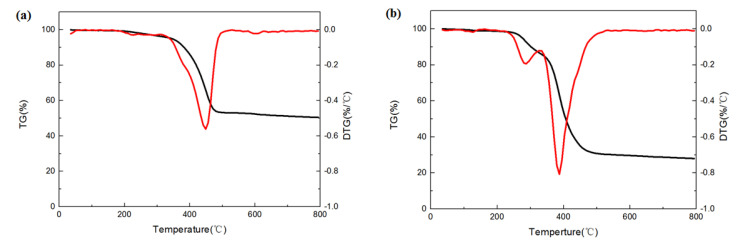
TGA (black lines) and DTGA (red lines) curves of WRP (**a**), HRP (**b**).

A thermogravimetric analysis measurement was used to study the thermal stability of WRP and HRP_7-15_. The obtained thermograms are shown in Figure 5. For the WRP, the slight weight loss in the TG curve at the beginning includes the escape of water and other organic additives added in the production process [40]. After that, it shows the main weight-loss zone where the WRP thermally decomposed. When the temperature rises to about 300 °C, the WRP begins to pyrolyze. It can be seen from DTG that at 448.8 °C, the maximum weight loss rate of WRP pyrolysis occurs, and the constant weight appears at about 530 °C indicating that the pyrolysis part of WRP has been transformed. Different from WRP, the DTG of the HRP has two decomposition peaks, one of which is at 284.9 °C. This is similar to the decomposition temperature of PAM, which proves that the main weight loss at this stage comes from the decomposition of the PAM grafted onto the surface of the rubber powder. The second decomposition peak appears at 388.6 °C, which is the decomposition of rubber network. This result indicates that the grafted PAM decreases the decomposition temperature of the WRP. In addition, the residue percentage of the HRP is 27.2%, much lower than the 51.1% for WRP. These results prove that PAM was successfully grafted onto the surface of rubber powder.

**Figure 6:**
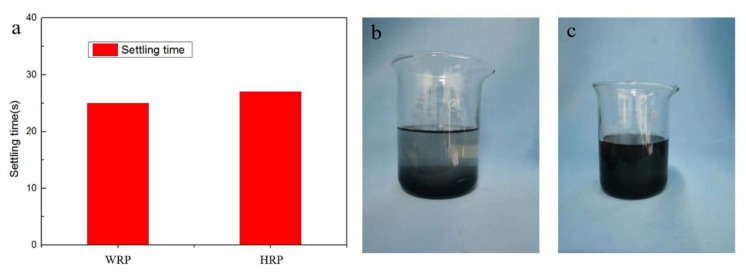
The setting time of WRP and HRP **(a**) and the photo of HRP solution after 4 h with suspension agent (**b**) and without (**c**).

We put the filler into the water and observed that the suspension of HRP_7-15_ or WRP settled in a short period of time. The settling time is shown in the Figure 6a. Because WRP has a relatively large particle size, even if it undergoes hydrophilic modification, it still precipitates in water, as shown in Figure 6b. Therefore, linear PAM is selected as the suspending agent. It can be seen that after adding PAM with a mass fraction of 1%, the rubber powder remains suspended after 4 h, as shown in Figure 6c.

**Figure 7:**
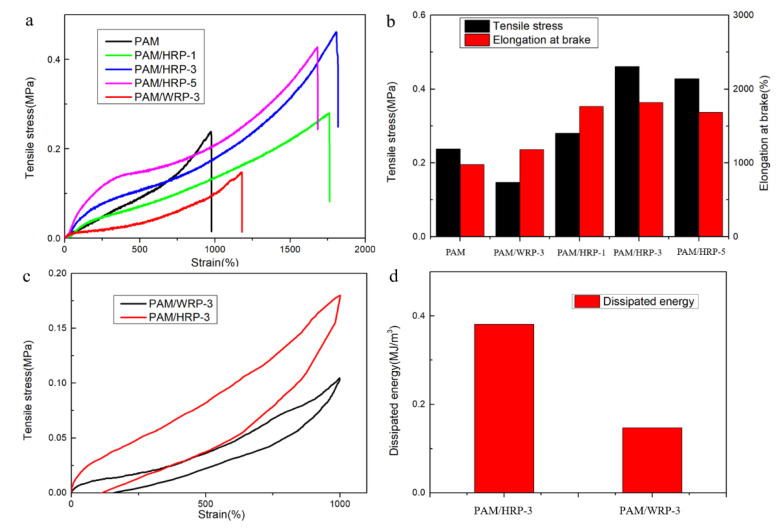
Tensile properties of the PAM/HRP hydrogels (**a**). The tensile strength and fracture strain with different PAM/HRP (**b**). The loading–unloading tensile test of PAM/WRP-3 and PAM/HRP-3 (**c**). The dissipation energy of PAM/HRP-3 and PAM/WRP-3 at 1000% strain (**d**).

To investigate the effect of HRP on the mechanical properties of composite hydrogels, tensile tests were conducted on PAM/HRP-x, in which the HPR_7-15_ was selected as a filler to be introduced into the hydrogel, and x was the mass of the HRP. In Figure 7a, all of the PAM/HRP hydrogels have much higher mechanical properties than the pure PAM hydrogel. Additionally, the tensile strength and the elongation at the break of the composite hydrogel increases first and then decreases with the increase of HRP. Among them, PAM/HRP-5 has the highest mechanical properties. The tensile strength and elongation at the break are 0.46 MPa and 1809%, which are 1.92 times and 1.85 times that of PAM hydrogel. Therefore, the addition of HRP has an evident strengthening effect on the hydrogel. It is worth noting that due to the lack of an interfacial interaction between the WRP and the matrix, the mechanical properties of PAM/WRP-3 are far inferior to those of PAM/HRP-3. The loading–unloading tensile test was used to study the energy dissipation mechanism of PAM/HRP-3. As shown in Figure 7c, the hysteresis circle of PAM/HRP-3 is larger than that of PAM/WRP-3. The dissipation energy of PAM/HRP-3 at 1000% strain can reach 0.38 MJ/m^3^, while the dissipation energy in PAM/WRP-3 is only 0.14 MJ/m^3^. Compared to PAM/WRP-3, the extra dissipation energy of PAM/HRP-3 is mainly contributed by the physical entanglement between HRP and the hydrogel network. These results show that PAM/HRP has sexcellent mechanical properties not only as a filler reinforcement effect of the rigid rubber particles but also for physical entanglement formed between the PAM chains grafted onto the surface of the HRP. The matrix limits the movement of the polymer chain and makes it difficult to break. However, adding too much HRP not only reduces the cross-linking density but also causes aggregation to cause stress concentration, thereby reducing the strength of the hydrogel.

To verify this hypothesis, the swelling properties and crosslinking density of PAM/HRP hydrogels were tested. As shown in Table 3, with the increase of HRP, the swelling ratio of the composite hydrogel gradually increases, and the crosslinking density decreases. Pure PAM hydrogel has the highest crosslink density, and PAM/HRP-5 has the lowest crosslink density. On the one hand, this may be due to the processing additives added during the tire manufacturing process remaining in the rubber powder [41]. These impurities additives can hinder the polymerization or crosslinking reactions during the synthesis of the hydrogel matrix. On the other hand, the steric hindrance of HRP particles also affects the crosslinking degree of the hydrogel. Therefore, the more HRP is added, the higher the impurity content is and the lower the crosslinking density is. The moderate addition of HRP is essential to improve the strength of the hydrogel.

**Figure 8:**
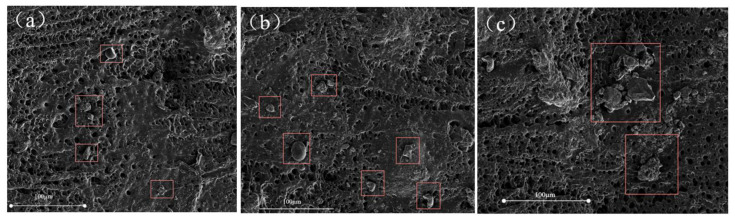
SEM images of composite hydrogels: PAM/HRP-1 (**a**), PAM/HRP-3 (**b**) and PAM/HRP-5 (**c**).

SEM observation was used to further analyze the influence of the dispersion of HRP particles in the PAM matrix at the microscopic scale. As shown in Figure 8, the surface morphology of PAM/HRP-1, PAM/HRP-3 and PAM/HRP-5 hydrogel samples were observed using SEM. For PAM/HRP-1 and PAM/HRP-3, due to the small amount of the addition, HRP can be dispersed relatively uniformly without obvious aggregation. When the mass of the HRP increased to 5 g, the aggregation phenomenon appeared, which caused stress concentration and reduced the mechanical properties of the hydrogel.

**Figure 9:**
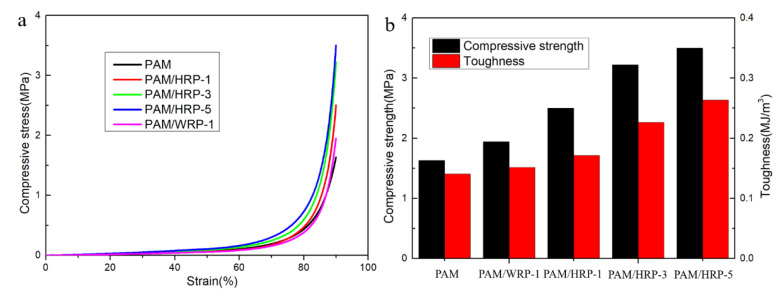
Compressive properties of the PAM/HRP hydrogels (**a**). The compressive strength and toughness with different PAM/HRP (**b**).

Similarly, the PAM/HRP hydrogel showed good compression properties in the compression tests. In the Figure 9, when the amount of HRP increased, the compressive strength of the hydrogel increased from 2.49 MPa to 3.51 MPa, which is higher than the traditional PAM hydrogel. Additionally, when the external force was removed, the deformation of the hydrogel could be completely recovered, showing good elasticity. Compared to pure PAM hydrogel, PAM/WRP-1 has higher compressive strength due to the filler reinforcement effect. The poor compatibility between WRP and matrix makes its mechanical properties inferior to PAM/HRP-1. The results show that the hydrophilic modification of WRP can improve the mechanical strength of the composite hydrogel.

## 3. Materials and Methods

### 3.1. Materials

Acrylamide (AM), Dibenzoyl peroxide (BPO), and toluene were purchased from the Chengdu Huaxia Chemical Reagent Factory. *N,N*-methylenebis (acrylamide) (MBA) was purchased from the Chengdu Kelong Chemical Reagent Factory. Potassium persulfate (KPS) and tetramethylenediamine (TEMED) were supplied by the Tianjin Bodi Chemical Industry Ltd., Tianjin, China. Waste rubber powders (WRP, 120 sieves) were from the Qingdao Huishang Rubber Company (Qingdao, China). In this study, the distilled water was used, which was made in the laboratory.

### 3.2. Preparation of Hydrophilic Modified Rubber Powder (HRP)

A total of 4 g WRP and 7 g BPO were added into 100 g toluene, and after magnetic stirring for 36 h, the solvent was removed. The treated WRP and 100 g distilled water were then added into a three-necked flask. Nitrogen was blown into the flask for 30 min to remove the influence of oxygen. A total of 15 g AM was added, and the temperature was raised to 65 °C for 1.5 h. The product gradually became viscous. Unreacted AM was removed from the product by washing with water. Finally, the product was dried at 40 °C to remove the water until a constant weight was reached. The final rubber powder that we obtained was called HRP.

### 3.3. Fabrication of PAM/HRP Hydrogel (PAM/HRP)

PAM/HRP hydrogel was synthesized by in situ free radical polymerization in an aqueous solution. AM, MBA, TEMED, and a certain amount of HRP were added to 100 mL deionized water until the HRP was dispersed in the water. A total of 1 g PAM was then added into beaker as suspending agent. After completely dissolving, the resulting suspension was poured into a single-necked flask and a vacuum pump was used to remove excess oxygen. The solution with the initiator KPS was added to the suspension. After mixing well, the suspension was transferred to the preformed mold and reacted at 25 °C for 4 h to obtain the PAM/HRP hydrogel.

### 3.4. Characterizations of Hydrophilic Modified Rubber Powder (HRP)

The conversion and grafting ratio of the HRP were calculated [42,43]. The weight of the WRP was W_1_, the weight of added AM was W_2_, and the weight of the product was W_3_. The equation of the yield and grafting ratio is expressed as:

(1) $$Conversion=\frac{W_{3}-W_{1}}{W_{2}}$$

(2) $$Graftingratio=\frac{W_{3}-W_{1}}{W_{1}}$$

FT-IR spectra of the functional groups on the surface of the rubber powder were recorded on a Nicolet-is50 infrared spectrometer (Thermo Fisher, New York, NY, USA) in the range of 4000–400 cm^−1^ with a 2 cm^−1^ resolution using a KBr pellet.

The impact of the different AM and BPO qualities on the hydrophilicity of the modified rubber powder was measured using the water contact angle. After the samples were pressed into a tablet, the contact angles of the samples were tested using a DSA25 Contact Angle Meter instrument (Kruss, Berlin, Germany). Each sample was tested three times, and the average value was taken as the result.

Thermogravimetric analyses of the modified rubber powder were tested using a TGA2 Thermal Gravimetric Analyzer (Mettler Toledo, Bern, Switzerland). The sample was heated from 35 °C to 800 °C at the rate of 10 °C/min under a nitrogen atmosphere. 

### 3.5. Mechanical Tests

All mechanical performance tests were conducted on a Instron 5567 material testing machine (Instron, New York, NY, USA). For tensile tests, the sample was cut into dumbbell models with a cross-section area of 4 mm × 1 mm. The stretching rate was controlled to 100 mm/min to record the stress–strain curve. For the cyclic tensile test, the tensile rate was unchanged, and the maximum strain was 500%. The dissipated energy generated by each sample during the stretching cycle was calculated from the area between the loading–unloading cycle curve. For the compressive tests, the cylindrical sample with a diameter of 13 mm was cut into a column with a height of about 20 mm. The compression rate was set to 5 mm/min.

### 3.6. Swelling Measurements of the PAM/HRP Hydrogel

The hydrogel samples were dried to a constant weight at 70 °C, and the gels were then swelled in excess distilled water at room temperature. The gel was removed every day to record the quality changes. The Flory–Rehner equation was used to calculate the crosslink density [44,45]. The equation is expressed as:

(3) $$\left[In\left(1-\varphi_{p}\right)+\varphi_{p}+\chi \varphi_{p}{}^{2}\right]=V_{0}n\left[\varphi_{p}{}^{\frac{1}{3}}-\frac{\varphi_{p}}{2}\right]$$

where *φ*_p_ is the volume fraction of the swollen polymer, χ is the Flory-Huggins parameter, and the value between the water and PAM hydrogel is approximately 0.44. V_0_ is the molar volume of the solvent (water is 18.054 cm^3^/mol), and n is the number of movable cross-linked segments per unit volume, which is the crosslink density. Based on Equations (4)–(6), we convert the obtained mass fraction into a volume fraction.

(4) $$\varphi_{s}=\frac{w_{s}V_{s}/V_{P}}{1-w_{s}\left(1-V_{s}/V_{P}\right)}$$

(5) $$w_{s}=M_{s}/\left(M_{s}+M_{P}\right)$$

(6) $$\varphi_{p}=1-\varphi_{s}$$

where V_P_ = 0.681 cm^3^/g, V_s_ = 1.0021 cm^3^/g, W_S_ is the mass fraction of the solvent at equilibrium swelling, M_S_ is the mass of the solvent in the swelling gel, M_P_ is the mass of polymer, *φ*_s_ is the volume fraction of solvent in swollen gel, and *φ*_p_ is the volume fraction of the polymer.

### 3.7. Characterization of Hydrogel Morphology

The hydrogel was put in the freeze dryer for 48 h to remove excess water. After brittle fracturing with liquid nitrogen, the cross section was sprayed with gold and its morphology was observed with the scanning electron microscope (SEM, FEI Inspect F, Hillsboro, OR, USA).

## 4. Conclusions

In this study, the hydrophilic modification was successfully realized by grafting PAM chains onto the surface of rubber powder. It was introduced into the hydrogel to prepare a novel PAM/HRP composite hydrogel. The SEM results show that the modified rubber powder can be uniformly dispersed in the PAM matrix. In addition to the filler enhancement effect, the physical entanglement formed by the PAM chains on the surface of the HRP and the hydrogel matrix endowed the composite hydrogel with an excellent energy dissipation mechanism, which effectively improved its mechanical properties. Among them, the tensile strength and the elongation at the break of PAM/HRP-5 reached 0.42 MPa and 1806%, which are 1.92 times and 1.85 times that of pure PAM, respectively. This work recycles waste rubber powder to reduce environmental pollution and provides a simple method to prepare a high-performance PAM hydrogel, which has broad application prospects in the petroleum industry and in agriculture. Such a strategy can be easily extended to graft different molecular chains onto the surface of waste rubber powder to achieve different functional modifications.

## Figures and Tables

**Table 1:** The grafting ratio of HRP_7-y_ with different amounts of AM.

	HRP_7-5_	HRP_7-10_	HRP_7-15_	HRP_7-20_
Total mass of product	5.55g	8.42g	12.34g	13.27g
The conversion of HRP_7-y_	31.0%	44.2%	55.2%	46.3%
Grafting ratio	38.7%	110.5%	208.5%	231.7%
Contact angle	101.1°	73.5°	59.6°	55.8°

**Table 2:** The grafting ratio of HRP_x-15_ with different amounts of BPO.

	HRP_3-15_	HRP_5-15_	HRP_7-15_	HRP_9-15_
Total mass of product	6.74g	10.94g	12.34g	12.95g
The conversion of HRP_7-y_	18.3%	46.3%	55.2%	59.7%
Grafting ratio	68.5%	173.5%	208.5%	223.7%
Contact angle	109.3°	8.88°	59.6°	53.6°

**Table 3:** The swelling property of PAM and PAM/HRP hydrogels.

Samples	M_p_ (g)	M_se_ (g)	M_s_ (g)	Swell Ratio	n (mol/m^3^)
PAM	1.0625	92.8069	91.7444	86.3476	1.0853
PAM/HRP-1	1.1142	102.2738	101.1596	90.7912	0.9913
PAM/HRP-3	1.1784	141.1174	139.9390	118.7533	0.6338
PAM/HRP-5	1.0838	177.1855	176.1017	162.4855	0.3768
PAM/HRP-7	0.8553	172.5214	171.6661	200.7086	0.2633

## Data Availability

The data presented in this study are available upon request from the corresponding author.

## Sample Availability

Samples of the compounds are available from the authors.

## References

[1] Zedler Ł., Przybysz-Romatowska M., Haponiuk J., Wang S., Formela K. (2020). Modification of Ground Tire Rubber—Promising Approach for Development of Green Composites. J. Compos. Sci..

[2] Zanchet A., de Sousa F.D.B. (2020). Elastomeric Composites Containing SBR Industrial Scraps Devulcanized by Microwaves: Raw Material, not a Trash. Recycling.

[3] De D., Maiti S., Adhikari B. (2000). Reclamation and recycling of waste rubber. Prog. Polym. Sci..

[4] Mohajerani A., Burnett L., Smith J.V., Markovski S., Rodwell G., Rahman M.T., Kurmus H., Mirzababaei M., Arulrajah A., Horpibulsuk S. (2000). Recycling waste rubber tires in construction materials and associated environmental considerations: A review. Resour. Conserv. Recycl..

[5] Estagy S., Ansarifar A., Movahed S.O. (2016). Review of the Reclaiming of Rubber Waste and Recent Work on the Recycling of Ethylene–Propylene–Diene Rubber Waste. Rubber. Chem. Technol..

[6] Yi F., Maosheng Z., Ying W. (2001). The status of recycling of waste rubber. Mater. Des..

[7] Fazli A., Rodrigue D. (2020). Waste Rubber Recycling: A Review on the Evolution and Properties of Thermoplastic Elastomers. Materials.

[8] Eldho A., Bibin M.C., Elbi P.A., Laly A.P., Sabu T. (2011). Recent advances in the recycling of rubber waste. Prog. Rubber Plast. Recycl. Technol..

[9] Marvin M., Sitisaiyidah S., Wilma D., Jacques N. (2012). Rubber recycling chemistry processing and applications. Rubber. Chem. Technol..

[10] Sun Y., Yan X., Liang H., Bohm G., Jia L. (2020). Rubber Recycling: Mending the Interface between Ground Rubber Particles and Virgin Rubber. ACS Appl. Mater. Interfaces.

[11] Moll J.F., Akcora P., Rungta A., Gong S., Colby R.H., Benicewicz B.C., Kumar S.K. (2011). Mechanical Reinforcement in Polymer Melts Filled with Polymer Grafted Nanoparticles. Macromolecules.

[12] Akcora P., Liu H., Kumar S.K., Moll J., Li Y., Benicewicz B.C., Schadler L.S., Acehan D., Panagiotopoulos A.Z., Pryamitsyn V. (2009). Anisotropic self-assembly of spherical polymer-grafted nanoparticles. Nat. Mater..

[13] Wu F., Zhang B., Yang W., Liu Z.Y., Yang M.B. (2014). Inorganic silica functionalized with PLLA chains via grafting methods to enhance the melt strength of PLLA/silica nanocomposites. Polymer.

[14] Luo M., Liao X., Liao S., Zhao Y. (2013). Mechanical and dynamic mechanical properties of natural rubber blended with waste rubber powder modified by both microwave and sol-gel method. J. Appl. Polym. Sci..

[15] El-Nemr K.F., Khalil A.M., Fathy E.S. (2017). Thermoplastic elastomers based on waste rubber and expanded polystyrene: Role of devulcanization and ionizing radiation. Int. J. Polym. Anal. Charact..

[16] Gupta V.K., Nayak A., Agarwal S., Tyagi I. (2014). Potential of activated carbon from waste rubber tire for the adsorption of phenolics: Effect of pre-treatment conditions. J. Colloid Interface Sci..

[17] Han H., Zeng X., Muhammad Y., Li J., Yang J., Yang S., Wei Y., Meng F. (2019). Preparation of Octadecyl Amine Grafted over Waste Rubber Powder (ODA-WRP) and Properties of Its Incorporation in SBS-Modified Asphalt. Polymers.

[18] Yu R., Gong Z., Guo W., Zhang H., Liu C. (2016). A novel grafting-modified waste rubber powder as filler in natural rubber vulcanizates. J. Appl. Polym. Sci..

[19] Liu H., Wang X., Jia D. (2020). Recycling of waste rubber powder by mechano-chemical modification. J. Clean. Prod..

[20] Lei G., Dejun L., Donghui R., Lianen Q., Wenchao W., Kuanfa H., Xiurui G., Tianchi C., Jingyao S., Chuansheng W. (2021). Effectiveness of original additives in waste rubbers for revulcanization after reclamation with a low-temperature mechanochemical devulcanization method. J. Clean. Prod..

[21] Araujo-Morera J., Verdugo-Manzanares R., González S., Verdejo R., Lopez-Manchado M.A., Hernández Santana M. (2021). On the Use of Mechano-Chemically Modified Ground Tire Rubber (GTR) as Recycled and Sustainable Filler in Styrene-Butadiene Rubber (SBR) Composites. J. Compos. Sci..

[22] Liu J., Liu P., Zhang X., Lu P., Zhang X., Zhang M. (2016). Fabrication of magnetic rubber composites by recycling waste rubber powders via a microwave-assisted in situ surface modification and semi-devulcanization process. Chem. Eng. J..

[23] Du M., Guo B., Jia D. (2005). Effects of Thermal and UV-induced Grafting of Bismaleimide on Mechanical Performance of Reclaimed Rubber/Natural Rubber Blends. J. Polym. Res..

[24] Wang X., Du J., Zhang X., Jia D. (2018). Mechano-Chemical Modification of Waste Rubber Powder with 2-Mercatobenzothiozole and 3-Aminopropyltriethoxysilane. Macromol. Res..

[25] Wan Y., Bu Y., Liu J., Yang J., Cai W., Yin Y., Xu W., Xu P., Zhang J., He M. (2018). pH and reduction-activated polymeric prodrug nanoparticles based on a 6-thioguanine-dialdehyde sodium alginate conjugate for enhanced intracellular drug release in leukemia. Polym. Chem..

[26] Han Z., Wang P., Mao G., Yin T., Zhong D., Yiming B., Hu X., Jia Z., Nian G., Qu S. (2020). Dual pH-Responsive Hydrogel Actuator for Lipophilic Drug Delivery. ACS Appl. Mater. Interfaces.

[27] Ding L., Li J., Wu C., Yan F., Li X., Zhang S. (2020). A self-assembled RNA-triple helix hydrogel drug delivery system targeting triple-negative breast cancer. J. Mater. Chem. B.

[28] Teng C., Lu X., Ren G., Zhu Y., Wan M., Jiang L. (2014). Underwater Self-Cleaning PEDOT-PSS Hydrogel Mesh for Effective Separation of Corrosive and Hot Oil/Water Mixtures. Adv. Mater. Interfaces.

[29] Rejane A.B., Paula J.P.E., Jullyana D.S.S.Q., Mayanna M.F., Miguel Â.C., José A.T., Juliana C. C. (2019). Hydrogel as an alternative structure for food packaging systems. Carbohydr. Polym..

[30] Song J., Chen S., Sun L., Guo Y., Zhang L., Wang S., Xuan H., Guan Q., You Z. (2020). Mechanically and Electronically Robust Transparent Organohydrogel Fibers. Adv. Mater..

[31] Shang J., Le X., Zhang J., Chen T., Theato P. (2019). Trends in polymeric shape memory hydrogels and hydrogel actuators. Polym. Chem..

[32] Lei Z., Wang Q., Wu P. (2017). A multifunctional skin-like sensor based on a 3D printed thermo-responsive hydrogel. Mater. Horiz..

[33] Li Y., Liu S.J., Chen F.M., Zuo J.E. (2019). High-Strength Apatite/Attapulgite/Alginate Composite Hydrogel for Effective Adsorption of Methylene Blue from Aqueous Solution. J. Chem. Eng. Data.

[34] Wang Y., Zhang J., Qiu C., Li J., Cao Z., Ma C., Zheng J., Huang G. (2018). Self-recovery magnetic hydrogel with high strength and toughness using nanofibrillated cellulose as a dispersing agent and filler. Carbohydr. Polym..

[35] Nor J.M.S., Ahmad F.A.I., Rohana A., Mohd H.H., Takaomi K. (2019). Enhancement of poly (vinyl alcohol) using delaminated layered double hydroxide for the formulation of mechanically strong nanocomposite hydrogel. J. Appl. Polym. Sci..

[36] Hu X., Liang R., Li J., Liu Z., Sun G. (2019). strong hydrogels achieved by designing homogeneous network structure. Mater. Des..

[37] Liu Q., Dong Z., Ding Z., Hu Z., Yu D., Hu Y., Abidi N., Li W. (2018). Electroresponsive Homogeneous Polyelectrolyte Complex Hydrogels from Naturally Derived Polysaccharides. ACS Sustainable Chem. Eng..

[38] Xia S., Song S., Gao G. (2018). Robust and flexible strain sensors based on dual physically cross-linked double network hydrogels for monitoring human-motion. Chem. Eng. J..

[39] Chen Q., Yan X., Zhu L., Chen H., Jiang B., Wei D., Huang L., Yang J., Liu B., Zheng J. (2016). Improvement of Mechanical Strength and Fatigue Resistance of Double Network Hydrogels by Ionic Coordination Interactions. Chem. Mater..

[40] Stephen D.S., Md M.U., Chanchal K.R., Md J.H., Majharul I.S., Md S.A. (2020). Mechanically tough and highly stretchable poly (acrylic acid) hydrogel cross-linked by 2D graphene oxide. RSC Adv..

[41] Wang Y., Huang G., Zheng J. (2016). Mechanochemistry modified waste rubber powder and its application in hydrogel. J. Polym. Res..

[42] Wu Y., Zheng Y., Yang W., Wang C., Hu J., Fu S. (2005). Synthesis and characterization of a novel amphiphilic chitosan-polylactide graft copolymer. Carbohydr. Polym..

[43] Wang L., Shen J., Men Y., Wu Y., Peng Q., Wang X., Yang R., Mahmood K., Liu Z. (2015). Corn starch-based graft copolymers prepared via ATRP at the molecular level. Polym. Chem..

[44] Cao Z., Wang Y., Wang H., Ma C., Li H., Zheng J., Wu J., Huang G. (2019). Tough, ultrastretchable and tear-resistant hydrogels enabled by linear macro-cross-linker. Polym. Chem..

[45] Alonso de Mezquia D., Doumenc F., Bou-Ali M.M. (2012). Sorption Isotherm, Glass Transition, and Diffusion Coefficient of Polyacrylamide/Water Solutions. J. Chem. Eng. Data.

